# Unpacking the implementation climate in general education settings in public schools: a sequential-explanatory mixed-methods study

**DOI:** 10.1186/s43058-025-00810-0

**Published:** 2025-11-28

**Authors:** Aksheya Sridhar, Alice Bravo, Yasmín Landa, Priyanka Ghosh Choudhuri, Wendy Shih, Olivia Michael, Jill Locke

**Affiliations:** 1 University of Washington, Seattle, WA 98115 USA; 2 University of California, Los Angeles, CA 90025 USA; 3 University of Miami, Coral Gables, FL 33146 USA; 4 University of California, Box, 951521, Los Angeles, CA 90095 USA

**Keywords:** Implementation climate, Evidence-based practices, School, Autism

## Abstract

**Background:**

Autism has significantly increased in the United States with 1 in 31 children affected and increasingly included in general education settings as mandated by the Individuals with Disabilities Education Act. While several evidence-based practices (EBPs) effectively support autistic students, organizational-level factors hinder successful implementation in school settings. Implementation climate–implementers’ collective perceptions that their organization prioritizes, rewards, and supports EBP implementation–strongly predicts EBP use. However, little is known about implementation climate within general education settings in public schools.

**Methods:**

A sequential explanatory mixed-methods approach was applied. Participants included 361 general education teachers (*n* = 161), special education teachers (*n* = 138), and paraeducators (*n* = 62) across 60 elementary schools. All participants served at least one autistic student included in a general education classroom, with the total number of autistic students served ranging from 1 to 30 (*M* = 3.75). Participants completed the School Implementation Climate Scale (SICS) and aggregate mean scores for each subscale (i.e., focus on EBP, education support, recognition for EBPs, rewards for EBPs, use of data to support EBPs, existing support to deliver EBPs, integration of EBPs) were calculated. To better understand EBP use, participants were randomly selected (*n* = 82: 24 general education teachers, 49 special education teachers, 9 paraeducators) to complete 30–40 min semi-structured interviews, with equal distributions of non, low, and high EBP users. Data were collected between 2021–2024.

**Results:**

Quantitative analysis via aggregate mean scores on the SICS (Total *M* = 1.8) subscales revealed that educators rated implementation constructs relatively low. Qualitative findings provide additional insights into implementation challenges such as limited educational and professional development opportunities, insufficient EBP preparation time, lack of resources for autistic children, and minimal recognition and reward for EBP implementation.

**Conclusions:**

Findings reveal a suboptimal implementation climate within general education settings in public schools for supporting autistic students. These insights suggest the need to improve the implementation climate to facilitate successful EBP implementation. Recommendations and key areas for future research are discussed. Limitations, including the need to examine the impact of outer contextual factors are described.

**Supplementary Information:**

The online version contains supplementary material available at 10.1186/s43058-025-00810-0.

Contributions to the Literature

- A supportive implementation climate (i.e., whether staff feel their organization values and supports an intervention) can increase the likelihood that educators use evidence-based practices (EBPs) for autistic students, who are being increasingly included in general education settings.
- Findings show that general education environments have poor implementation climates for supporting autistic students. Challenges included limited training and professional development, insufficient EBP preparation time, lack of resources for autistic students, and minimal recognition or rewards for EBP use.
- Future research is needed to explore strategies to strengthen support for educators by improving how schools value, encourage, and promote EBP implementation.

## Background

Autism is a neurodevelopmental condition that affects social communication and interaction, while also involving repetitive behaviors and restricted interests [1]. The prevalence of autism has risen significantly in recent years, with current estimates indicating that 1 in 31 children in the United States are autistic [2]. This surge poses unique challenges for public schools, which are tasked with providing a free and appropriate education for all students [3]. Many autistic students are educated in general education settings, either partially or fully included alongside neurotypical peers (i.e., students without autism or other developmental differences). However, ensuring that autistic students receive effective educational support is complicated by systemic barriers and the variability in adoption and sustainment of evidence-based practices (EBPs) in schools [4–6].

### Individuals with disabilities education act

In the United States, a key legal framework governing the education of students with disabilities is the Individuals with Disabilities Education Act (IDEA, 3), which mandates that students with disabilities, including autism, be educated in their “least restrictive environment” (LRE)—that is, in settings that allow for the greatest possible access to learning alongside their neurotypical peers. The specific LRE setting is individualized and varies based on student support needs, ranging from full inclusion in a general education setting to receiving specialized programming outside of the school district. Regardless of placement, the emphasis remains on maximizing opportunities for participation in academic, social, and extracurricular activities with one’s same-aged peers and minimizing segregation. However, the reality of actualizing LRE and providing effective educational services in inclusive settings often is hindered by gaps in school resources, staff training, and ongoing support for educators [7, 8].

### Evidence-based practices

While there is a growing body of research identifying EBPs that are effective for supporting autistic students, such as visual supports, modeling, and reinforcement strategies [9], the successful implementation of these practices in school settings remains inconsistent [6, 10]. Educators often face challenges in using EBPs due to inadequate training, insufficient school resources, and a lack of ongoing administrative support [11]. As a result, even when educators adopt EBPs, they often struggle to achieve and maintain fidelity [12]. This is particularly concerning for students receiving special education services, as inconsistent or low-fidelity implementation of EBPs can lead to limited progress. Autistic students often have individualized academic and behavioral support needs [13], and inconsistent implementation of EBPs selected to address those needs can diminish their effectiveness and prevent students from fully benefiting from the intended support [14]. As such, promoting high-quality fidelity of EBPs is not just best practice—it is essential for equitable access to the appropriate public education students are promised under IDEA [3]. This underscores the need for more effective implementation strategies to support the sustainable, high-quality implementation of EBPs in schools.

To address these challenges, research must move beyond identifying which practices are effective and toward understanding how to support their consistent use in educational settings. Implementation science offers useful frameworks for studying the conditions that influence whether EBPs are adopted, implemented with fidelity, and sustained over time. In particular, exploring school-level factors, such as the school’s implementation climate, that shape educators’ ability to deliver EBPs can provide important insights into what drives successful implementation.

### Implementation climate

Implementation climate is a particularly important area for supporting schools to implement EBPs for autistic students. Implementation climate refers to educators’ shared perceptions of how strongly their school promotes the use of EBPs and is typically measured through distinct constructs (e.g., existing supports, focus on EBP, use of data, see Table 2). Implementation climate reflects the extent to which staff perceive EBP use as expected, supported, and rewarded by their organization [15]. Studies consistently illustrate the relationship between implementation climate and various outcomes, such that positive implementation climate is associated with decreased staff burnout and turnover [16], high-quality intervention implementation and sustainment [17], improved implementation fidelity [18, 19], and better child outcomes [17].

Although implementation climate is integral to improving implementation-level outcomes, there is limited research exploring implementation climate within school settings [20]. Few studies have examined educator perspectives regarding implementation climate. One study found that higher intervention fidelity was associated with improved student outcomes in schools in classrooms where educators perceived a strong implementation climate [21]. These findings are similar to research in other contexts that highlights the relationship between implementation climate and implementation-level outcomes [16–18]. Based on survey research among educators and school administrators, higher implementation climate was found to predict improved implementation outcomes, particularly for educators with less direct experience working with autistic individuals [22]. Findings also revealed that school administrators and educators believe that implementation climate in educational settings could be improved. Previous research has indicated that specific constructs of implementation climate (i.e. Focus on EBP, which refers to the extent to which implementing an EBP is a priority within an organization; see Table 2) were rated higher within the education sector compared to other contexts, highlighting the possible variability of implementation climate in different settings [20]. However, there is limited knowledge of what implementation climate constructs look like within school settings, and therefore limited understanding of how to improve upon this key driver of implementation success. This may be particularly important as implementation efforts may require further support and a positive implementation climate that is conducive for successful implementation within the context of special education.

Research has demonstrated that implementation climate is a malleable determinant that can be targeted in schools to support the use of EBPs for autistic students [23]. Assessing the implementation climate within schools can provide valuable information regarding the extent to which a school is ready to implement and support the adoption and successful use of a new intervention, as well as highlight key areas for organizational change [24]. While previous research has examined implementation climate in schools quantitatively [20, 25], and some research has done so qualitatively [26], this paper will leverage mixed-methods design to combine both quantitative and qualitative data. Given the heightened stakes of special education services—where ineffective implementation can lead to segregation or inadequate support—this research will allow us to better unpack and understand what implementation climate looks like within school settings. Because implementation climate may influence how effectively schools deliver EBPs, it has the potential to play a critical role in meeting IDEA’s mandate to provide a free and appropriate public education in the least restrictive environment for autistic students. Findings will inform relevant implementation strategies that address organizational determinants such as implementation climate within educational settings [27].

### Study objective

The purpose of this mixed-methods study is to evaluate, describe, and integrate educator perspectives regarding constructs comprising implementation climate in general education settings in public schools. This work aims to inform future implementation strategies that promote effective use of EBPs to support autistic students in inclusive classroom settings, as part of a broader program of research focused on improving EBP implementation in schools serving autistic students.

## Method

### Study design

This study followed a sequential explanatory (quan → QUAL) mixed-methods design. Quantitative data were collected first, followed by qualitative data for the purpose of exploring and elaborating upon each implementation climate construct [28].

### Participants and recruitment

Following university Institutional Review Board and school approval processes, we contacted school district officials to identify elementary schools with partially or fully included autistic children in general education classrooms. To recruit a representative sample of kindergarten-fifth grade (K-5) educators that was generalizable to the surrounding region and broader United States demographics, a recruitment plan was developed using a free web-based tool (https://thegeneralizer.org). This tool creates a stratified recruitment plan based on demographic features of districts and generates a ranked lists of schools to contact as well as recruitment goals to guide the recruitment process. The target sample of 360 educators was selected to achieve adequate statistical power while reflecting a representative balance of school characteristics (size, percentage of students receiving free and reduced lunch, gender, race, and urbanicity/rurality) and district characteristics (number of schools, percentage of English Language Learners, primary home language, and urbanicity/rurality).

Following the stratified recruitment plan, recruitment materials were distributed to school principals, general education teachers, special education teachers, and paraeducators (school staff who assist teachers with instruction and student support). Participating educators were required to be currently working with at least one autistic student to ensure they had recent, relevant implementing EBPs with autistic students. This criterion ensured that all participating educators were actively working with at least one autistic student, so they could provide current information on their EBP use with autistic students. Although participation required working with at least one autistic student, educators reported working with an average of 3.75 autistic students (range: 1–30). Approximately 826 interest forms from school educators were received from educators interested in completing the survey. Of those who completed the interest form, 361 educators (43.7%) consented to participate in the study and completed the survey.

A total of 361 general education teachers (*n* = 161), special education teachers (*n* = 138), and paraeducators (*n* = 62) who serve at least one autistic student in a general education classroom were recruited across 60 public elementary schools. The recruitment goal was 360 educators to ensure a representative sample of perspectives that was generalizable to K-5 educators in the surrounding region and broader United States. As survey invitations were distributed on a rolling basis, one additional educator completed the survey, resulting in a final sample of 361. Of these, 82 participants (i.e., 24 general education teachers, 49 special education teachers and 9 paraeducators) were interviewed. See Table 1 for sample demographics.

**Table 1:** Participant demographics

Demographics	Mean	SD
Age	41.5	11.9
Years in Current Position	7.9	6.3
Years in SPED	8.8	5.6
	***n***	**%**
**Role**		
General Education Teacher	161	44.6%
Special Education Teacher	138	38.2%
Paraeducator	62	17.2%
**Gender**		
Female	317	87.8%
Male & Female	1	0.3%
Male & Non-Binary	3	0.8%
Male	37	10.3%
Non-Binary	1	0.9%
Prefer not to answer	2	0.6%
**LatinX**		
Mexican, Chicano	2	0.6%
Cuban	16	4.4%
Other	12	3.3%
Not Hispanic/LatinX	331	91.7%
**Race**		
Asian	11	3.0%
Black/African American	7	1.9%
White	311	86.2%
Other	8	2.2%
Multi	24	6.6%
**Degree**		
High School Diploma	10	2.7%
Associate’s Degree	17	4.7%
Bachelor’s Degree	72	19.9%
Master’s Degree	255	70.6%
Doctoral Degree	2	0.5%
Other	5	1.4%

### Quantitative data collection

Consented educators were asked to complete a survey which lasted approximately 60—75 min. Participants were provided with a $60 incentive for completing the survey. The survey consisted of a demographics questionnaire, their EBP use, and the School Implementation Climate Scale (SICS; 30) to measure implementation climate.

#### School Implementation Climate Scale (SICS)

The SICS is a 21-item rating scale, with each item being rated on a 0–4 scale (0 = not at all to 4 = very great extent) that was successfully adapted from the Implementation Climate Scale (ICS; [29] for use in schools. It measures collective perceptions of policies, practices, procedures, and behaviors school employees are expected, supported, and incentivized to adopt for facilitating effective EBP implementation. The SICS has seven main subscales: 1) focus on EBP, which refers to commitment of their use; 2) educational support for EBPs, regarding learning and using EBPs; 3) recognition for EBPs, such as praise and acknowledgement; 4) rewards for EBPs, including financial and tangible incentives; 5) use of data to support EBPs through process such as fidelity assessments and data-driven feedback; 6) existing supports for EBPs, like professional development time; 7) and EBP integration in areas such as performance evaluations. This measure is a psychometrically validated and reliable instrument (α = 0.81–0.90; 30). Definitions for each construct are included in Table 2 [30].

**Table 2:** School implementation climate scale constructs and definitions

SICS Construct	Definition
Rewards for EBP	How the organization provides financial compensation or benefits for EBP implementation
Use of Data	Shifts in schools to incorporate data-based decision-making processes and tools as a way of creating accountability and encouraging school staff to implement an EBP
Existing Supports	The extent to which staff perceived that the school provides support such as professional development, coaching, and structured meetings to help them learn about and apply an EBP
EBP Integration	The extent to which EBP is integrated with other school systems and processes, including improvement plans, performance evaluations, and other ongoing work
Recognition	How much an organization values and recognizes providers for EBP implementation (non-financial recognition)
Educational Supports	Amount of training and materials that are provided to support EBP implementation
Focus on EBP	The extent to which implementing EBPs is an organizational priority

*EBP refers to “Evidence-Based Practice”.* Thayer, A. J., Cook, C. R., Davis, C., Brown, E. C., Locke, J., Ehrhart, M. G., Aarons, G. A., Picozzi, E., & Lyon, A. R. (2022). Construct validity of the school-implementation climate scale. *Implementation research and practice*, *3*, 26,334,895,221,116,065. https://doi.org/10.1177/26334895221116065

During the survey, each participant also was asked to indicate their use of four EBPs: antecedent-based intervention (arranging events or circumstances before a demand to increase desired behaviors or reduce interfering ones), reinforcement (applying a consequence after a skill or response to increase its future use), visual supports (using visual displays to help learners engage in desired behaviors independently), and modeling (demonstrating a target behavior to promote its use and acquisition by the learner) [31]. These practices were included because they were the most frequently used EBPs by educators, as identified by Locke and colleagues, based on survey responses in a prior phase of the study [32]. If they indicated the use of an EBP in the survey, participants were prompted to complete a fidelity checklist that detailed the necessary steps for effective implementation of the EBP [31]. Following the completion of the survey, the research team assessed participants' fidelity to each EBP by calculating the ratio of steps followed to the total required steps for that practice. Participants who reported not using a specific practice were categorized as not applicable (NA) for that EBP. Those who indicated using an EBP but did not select any steps from the fidelity checklist were classified as non-users. Participants were considered low users if they completed 70% or less of the required fidelity steps for visual supports or reinforcement, 66% or less for modeling, or 40% or less for antecedent-based intervention. Participants who reported completing over 70% for visual supports and reinforcement, 88.3% or more of the steps related to modeling, and 80% or more for antecedent-based interventions were classified as high users. These fidelity step cutoffs were determined based on prespecified, study-specific criteria established by the research team. Participants were then categorized as non-users, low users, high users, or not applicable (NA) for each of the four practices.

### Qualitative data collection

#### Semi-structured interviews

Participants who were previously categorized as non, low, and high users through the quantitative survey were entered into a randomization table to determine who would be invited to a semi-structured interview. Each was assigned a primary and secondary EBP for consideration. The randomization table was designed to ensure an equal distribution of non, low, and high users across the four EBPs while accounting for an anticipated 25% dropout rate among interview invites. If a participant's usage status (e.g., non, low, high) matched the designated status for their primary EBP (e.g., antecedent-based intervention, visual supports, modeling, or reinforcement) in the randomization table, the participant was selected to be invited for a semi-structured interview. Those classified as NA for their primary EBP were excluded from the randomization process.

Selected participants were contacted via email and informed of their invitation to participate in an interview. If the invitation was accepted, an interview date was scheduled within 2–4 weeks of survey completion. The research team generated interview guides that outlined the order of questions for the interviewer, beginning with the primary and secondary EBPs from the randomization table and followed by the remaining two EBPs. Drawing from the Exploration, Preparation, Implementation, Sustainment framework, we systematically developed theoretically-informed questions. These examine individual (e.g., attitudes, norms, and self-efficacy) and organizational constructs (e.g., implementation leadership and climate), their mechanisms of action, and whether they serve as facilitators or barriers to teachers’ and paraeducators’ use of EBPs in general education settings to successfully include and retain autistic children.

Interviews were conducted by several members of the research team (i.e. research assistants, coordinators, postdoctoral trainees) and supervised by the senior author. Interviews took place via videoconferencing software and audio-recorded. Participants' interviews lasted approximately 30–40 min, and participants received a $40 incentive upon completion. A qualitative codebook was developed, and consensus coding was used to summarize implementation constructs and identify theme(s) related to implementation climate (See Supplemental Materials).

### Quantitative data analysis

Quantitative data analysis involved aggregating mean scores across all participants for each SICS subscale as well as the mean total SICS score.

### Qualitative data analysis

Qualitative data analysis began with transcribing and verifying interview data through research team transcript review. Participant roles (i.e. general education teacher, special education teacher, paraeducator) and randomly generated participant IDs that were developed during prior phases of the study were used in interviews to anonymize data. Coding was conducted using NVivo software (version 13.0) by two independent coders. Codes were generated both inductively (i.e. emergent codes) and deductively (i.e. codes were identified based on guiding implementation framework) following a thematic analysis approach [33]. The Consolidated Framework for Implementation Research (CFIR) framework [34] was used to organize the coding scheme by five categories: Inner Setting, Outer Setting, Individuals, Implementation Process, and Innovation Characteristics. Inductive codes (e.g. classroom placement, educator characteristics for EBP use) were then organized into one of the five CFIR domains. Coders reached interrater reliability (97% average percent agreement) on a subset of interviews and updated the coding scheme iteratively throughout the process. Following this, coders independently coded the remaining transcripts, and drift was calculated for 10% of the remaining interviews (average was 97% agreement). Both interrater reliability and coding drift were calculated using reliability features on the NVivo software. A consensus process was used to resolve any discrepancies. Upon completion of the coding process, both coders worked together to first summarize each code and organize qualitative data under topic summaries representing each implementation climate construct [35]. Next, coders reviewed each topic summary and defined one qualitative theme that represented participant’s responses.

### Mixed methods analysis

The first author then created a joint display (i.e. side by side comparison table; [36] to integrate the quantitative and qualitative data strands. Coders reviewed quantitative and qualitative data for each implementation construct and through consensus discussion, identified points where data strands converged or were consistent with one another, or diverged based on participant responses on each data strand.

## Results

### Quantitative results

Aggregate mean scores on the SICS (Total *M* = 1.8) subscales illustrate educators’ perceptions regarding implementation climate at their schools (Table 4). Results indicated low integration of EBPs (*M* = 1.9) but a moderate focus on implementing EBPs (*M* = 2.4) in school settings. Educators also reported low to moderate availability of existing support (*M* = 1.7) and educational support (e.g. training and materials; *M* = 2.0) for staff to learn about EBPs. Additionally, educators reported low use of data to support the use of EBPs (*M* = 1.6). Although staff reported a moderate level of recognition (*M* = 2.0) for using EBPs, they reported lower use of rewards (*M* = 0.7) for implementing EBPs in their schools.

### Qualitative results

Qualitative analyses provided a deeper understanding of each of the constructs that comprise implementation climate, based on educator perspectives. Two codes falling under the “Inner Setting” category were used to unpack implementation climate: school and resources. Coders identified one overarching theme that best represented the qualitative findings: Tailoring Implementation Processes. Qualitative findings consistently revealed the potential need for tailoring implementation strategies in an effort to improve the implementation climate within schools specifically. Topic summaries falling under this theme are presented in Table 3.

**Table 3:** Qualitative topic summaries and quotes

Implementation Climate Construct	Topic Summary	Illustrative Quotes
*Focus on EBP*	This construct represents the extent to which implementing EBPs is a priority within the organization. Educators highlighted (a) lack of focus on implementing EBPs within their schools. In particular, special education teachers and paraeducators expressed (b) lack of knowledge among general educators regarding the strategies used by the special education team, and believe that general education teachers need to better understand EBPs and how to use them for autistic students in their classes. Additionally, special education staff described (c) a separation of responsibilities across general and special education teachers and noted that general education teachers need to “take ownership for these students” given the push for greater inclusion of autistic students in general education settings	SpEd teacher: *“That's been a big push this year, someone is wanting to be a part of a team because this program is part of the whole school. So I need someone in administration that is going to support that and have an expectation that General Ed teachers own these kids. And so there's zero support”* SpEd teacher: ***“****I feel like there's a culture at this school where paras want to make their own choices and they don't want direction from teachers. I've had administrators say they're teachers, too. There's just this culture. Then, I guess it's a culture for teachers, too. I don't know if it's that they just don't understand, they haven't had the education behind it, so they don't understand it. Because the other classrooms don't work with general ed. I'm the only one that works with general ed.”*
*Educational Support*	Educational support refers to the amount of training and materials provided at the organization to support implementing an EBP. Several issues related to this construct were raised a) Few educators reported that they receive support at their schools and from leadership to use their time to attend training/professional development events and to create relevant materials (e.g. visuals), particularly at the beginning of the school year Responses related to educational support for EBP implementation were overwhelmingly related to a lack of support in the form of funding, access to tangible resources, professional development, and training/education for using specific EBPs b) Lack of funding was a salient barrier to implementation, and was typically discussed in conjunction with limited training opportunities and access to tangible resources. Educators explained that resources (e.g. sensory tools, reinforcers or rewards, materials to make laminated visuals) often are lacking. Educators often used their own funds to purchase materials, or relied on the special education budget if available. Educators relied on each other and shared resources needed for using EBPs, such as account access to programs used for creating visual supports c) Participants also explained that even when they have access to tangible resources, not all educators felt equipped to adapt or use these materials for autistic students. Indeed, educators reported a need for further training focused on autism and supporting autistic students in general education settings specifically, as trainings reportedly tended to focus on students in self-contained classrooms d) Educators also emphasized the need for support related to professional development and training for general education staff in particular, to increase knowledge and understanding of how best to support autistic students within general education settings. These responses were salient, as educators often expressed a sense that general education teachers are unfamiliar with autism intervention strategies, and general education teachers reported having less understanding of how to tailor EBPs to their autistic students. Additionally, both special and general educators have to advocate for themselves in order to attend professional development events; one educator stated, “unfortunately it’s really up to me to educate myself if I feel I need to do more for a student.” Overall, qualitative findings indicate a significant need for increasing access to educational support when implementing EBPs in inclusive settings	SpEd teacher: *“I'd say that they provide us with opportunities for trainings. Every beginning of the school year, there's lots of trainings on different things that we want to do as a building. There's opportunities for students. And I feel like if … This year, we reached out and we said, ‘Hey, we would like some more training for a para educator in [Included Student with Autism]'s room. We would like some autism training for the gen ed teachers who work with [Included Student with Autism]’ and they offered all those. So the support from administrators is good.”* GenEd teacher: *“So, any sort of tools that we try to bring into support kids are all on our own…Our special education budgets are very, very small to my knowledge. And we all try to do the best we can.”* SpEd teacher: *“I would love support in getting the tangible items. It's been a burden on us special education teachers to have to be providing all the items that we're reinforcing with. And then I would love to have maybe a brainstorming session would be fabulous with a behavior specialist or someone to try to find that thing for [Included Student with Autism] that she will want every single time.”* SpEd teacher: *“…every once in a while, our special education department will offer some special education specific professional development, but they don't offer a lot. And what they offer tends to be geared not towards students who are included in general education classrooms, but students who are in self-contained classrooms.”*
*Recognition for EBPs*	This construct highlights the extent to which an organization values and recognizes the individuals implementing an EBP. Qualitative analyses indicated three categories of responses related to recognition: a) educators who reported receiving primarily verbal recognition and feeling valued for their EBP implementation and efforts. These educators described the recognition they receive for implementing EBPs which includes verbal recognition from leadership and colleagues in team meetings, during live classroom observations, via email, through teacher appreciation events, and in meetings with students’ parents. Additionally, educators noted receiving prizes or “treats in the lunchroom” as a form of recognition. Overall, some educators reported a feeling that “everyone is valued” and that schools “use a lot of positive feedback.” b) educators who reported a greater need and desire for receiving recognition for using EBPs with autistic students. For example, these educators expressed wanting opportunities or a system in place to share about student growth and to receive positive reinforcement from leadership and their colleagues. One educator reflected that receiving more positive feedback would be welcomed c) A number of participants reported that they do not need or want recognition and that receiving recognition would make them feel awkward	SpEd teacher: *“Okay. So recognizing verbally in meetings with parents, sometimes she'll send an email saying, "Nice work," she's great about writing little notes a couple times a year, and she'll do that for everybody on staff, she'll put little notes in our boxes and just say in general what a good job we're doing… “* SpEd teacher: *“I see that you set up some really cool antecedent intervention for this kid,’ or any of the other specific practices that we do, I think would be really cool”* Paraeducator: *“sometimes we'll get praised if we use praise well”*
*Rewards for EBPs*	Rewards for EBPs refer to financial compensation and/or benefits provided by the organization for EBP implementation efforts. Overall, educators reported that schools do not provide rewards in the form of financial compensation or other benefits to educators for their EBP implementation efforts. Some educators also reported that they did not have a desire to earn rewards as they see EBP implementation as a part of their job responsibilities	SpEd teacher: *“Oh, she doesn't. I wouldn't expect her to either. I mean, recognize, we do little shout-outs at our staff meetings. And I would say something like my other student on the spectrum, I said the other day like, "Hey, this kid wrote a whole paragraph." And they're like, "Whoa, way to go." Because we all know him. And the fact that he did that on his own is great. It's a little bit of a boost. She knows our students fairly well, so [Included Student with Autism] does not need that intervention. If I needed to send a kid out or if I needed them to have a space to be supported, I could send them to the office and she would be able to help that. I guess that's kind of a tangent. But yeah, she rewards with a shout-out, rewards by trusting us to do our job, I guess, which I like.”*
*Use of Data to Support EBPs*	This construct represents changes in schools to use data-based decision-making processes and tools to create accountability and encourage staff to implement EBPs. Educators reported that they track student progress, and then share these data with administrators to illustrate success with EBP use. They also expressed that sharing these data with administrators increases the expectation that educators continue to use EBPs with their students. Additionally, educators reflected that leadership “do a lot of research” to understand EBPs, and review student charts and behavior intervention plans to inform decision-making processes around EBP use	GenEd Teacher: *“I guess the short answer there is that we put in the work. We get to know the students, we look at the data, the feedback, take feedback from parents, directly from students, and we've got a kick-ass counselor here who is just, I don't know how he meets with so many people in such little time, but I'm always in communication with the counselor too, and he was able to share more information to help me know what I can provide the students in order for them to be successful during whatever we're doing.”*
*Existing Supports to Deliver EBPs*	This construct represents staff perceptions that the school provides support for staff to learn about and implement an EBP a) Educators who reported that they do receive support for EBP implementation explained that leadership provides support by giving educators access to existing materials (e.g. colored printers to create materials, computer programs to access visual supports) and in some schools, allowing educators to use a resource budget to purchase additional materials. An additional form of support also included having access to specialists (e.g. autism coordinator, inclusion specialists, case managers) who provide educators with additional information and education around using EBPs with autistic students. While this form of support was reported by some educators, others described a need for specialized staff, and more staff in general, at their schools b) Most salient when discussing existing support for EBP implementation was a lack of time. Although educators have time set aside for staff meetings and to prepare their curricula, educators reported that this time was not reallocated in any way for time spent discussing or preparing for EBP implementation. Educators expressed having limited time to plan, prepare EBP materials, train colleagues and to track student data in response to EBPs. Relatedly, educators noted that greater communication and collaboration is needed to build out special education programming and support teachers in including classrooms. While some educators are given preparation time for their students more broadly (i.e. not for EBP implementation), these preparation times often do not overlap with other educators working with the same autistic students. As a result, educators have difficulty finding additional time to communicate and collaborate with other team members involved in the care of their autistic students. One educator suggested using existing structures (e.g., Microsoft Teams) to provide opportunities for communication within teams c) Participants also reported using time outside of school hours to prepare EBP related materials. Notably, educators noted that using an EBP such as modeling is more feasible than using more time-consuming EBPs such as visual supports and antecedent-based interventions. Educators explained that using visual supports requires greater preparation time to create materials, while antecedent-based interventions involve spending more time taking and reviewing student data d) Lastly, participants expressed that while school leaders provide support in the form of additional intervention ideas, lack of time continues to impede educators’ ability to learn more about the EBP and how to implement EBPs successfully	GenEd Teacher: *“So I feel like there could be a lot that's happening around this. I feel like there needs to be support for teachers that are inclusive classrooms. It's never really talked about. It's kind of like, oh, you have this group of 28 students and that's it. And there's not any digging deeper, like, especially online. There's just so many ways that we could be connecting with teachers, with children, with autism. Like we could be sharing what works. There could be teachers that have never experienced this before and don't even know where to start. And so I would love it if they could offer, you know, PD or some type of collaborative group that meets online or even a private channel or a private team in our Microsoft Team where we can help each other support. Because I mean, if I have three students, I know someone else does, but it's never talked about, you know.”* SpEd Teacher: *“Well, sometimes it's just having someone available to do that with him? Not having enough staff. If he's in gen ed and his teacher is helping other students, she can't always be there to do that for him. So that's really the hardest thing to overcome, I think. And sometimes I have staff to send with him, but not all the time.”* SpEd teacher: *“The factors that affect my plans and intentions would definitely be just a lack of time built into the schedule. And it's just a regular thing, I think, with most teachers, if your schedule is packed with teaching groups and monitoring and data collecting and writing IEPs and all those things, then you don't have quite as much time to plan how to implement some of those strategies that you would like to.”* GenEd teacher: *“It's like she gives us articles to read at staff meetings with interesting ideas in it, but at that point, it's just like, "Oh, these are great ideas, but where's the tools? Where's the time? Are you going to help us with this?" I leave the staff meetings like, "Oh, those are some creative ideas." Then I get in here and I'm like, "Oh shit. Now, I only have a half an hour to plan stuff." It's just like, "Okay, now, that's just something in my file folders here that hopefully I can get to soon and implement." Then at some point, I'm just like, "I would kind of rather just spend time with my family and go home."*
*Integration of EBPs*	Integration of EBPs refers to how much an EBP is integrated into existing school systems and processes a. Educators described ways in which three of the four EBPs discussed were integrated in their schools. Reinforcement, modeling, and visual supports were reportedly used school or classroom-wide for several educators, with several educators highlighting an expectation from leadership that they use their strategies with their students. Participants explained that reinforcement was widely supported by administration, and integrated relatively seamlessly into schools already using other reinforcement or positive behavior support systems. Some educators noted a similar school-wide expectation for using visual supports, while others stated that they used these strategies for all students in their classrooms. Lastly, educators at some schools reported that they had received school-wide training to use modeling with their students, and that both general and special education teachers were using these strategies. Although antecedent-based interventions were less integrated in schools currently, one educator noted an increased push for using this intervention in an effort to be more proactive in addressing challenging behaviors at school b. Conversely, educators also described ways in which EBPs are not well integrated into their schools. In some cases, educators reported that they were not aware of how to use EBPs and did not always follow through on EBP use. Additionally, educators highlighted that there may be a tendency to rely on using universal strategies that work well for the majority of students in general education settings, rather than integrating tailored strategies to use with autistic students specifically. Finally, similar to other implementation climate constructs, educators reported a need for increased buy-in from teachers, and specifically general education teachers, in order to better integrate EBPs into general education settings	SpEd teacher:* “I’m looking around the rooms right now and visuals everywhere.”* GenEd Teacher:* “Again, they expect us to use modeling in general. They want us to use modeling with all of our students, and so any way that we can mix it up, make it fun, make it interesting, make it engaging for all of our students, they're going to want us to do that so that we can reach as many kids as we can using as many ways possible that we can.”* GenEd Teacher:* “So it's kind of nonexistent, not in a mean way. I'm not going to say it's to be rude. I think it's more, just a lack of oversight as far as there are students that are in our classroom that need these specialized practices and there's not really follow through on it. I'm kind of doing it because I know it's the right thing to do and I care about it.”* Paraeducator:* “And so like I said, kind of our philosophy that the special ed teacher and myself is that less is more. We don't want to add on superficial things that aren't going to benefit him, like star charts or whatever”*

### Mixed methods results

A joint display was created to illustrate where quantitative and qualitative data converge and diverge (Table 4). Data are presented in order from low to moderate quantitative ratings, while qualitative data illustrate participant perspectives and rationales concerning each implementation climate construct.

**Table 4:** Joint display

**SICS Construct**	**Quantitative Strand** M (SD)	**Overall Rating**	**Qualitative Strand** Illustrative Quote	Data Strands Converge/Diverge
Rewards for EBP	0.7 (0.8)	Low	*“I don't think I would want to be rewarded. That was one of the things that you… I don't think I should be rewarded for learning more best practices or whatever. I think that's just part of the job.”* [GenEd teacher]	Converged
Use of Data	1.6 (0.8)	Low	*“Now again, our focus and discussions are around if a student is struggling, what data and interventions are you using? And so a lot of that is looking at what's happening for the student, and that our expectation is that we as a staff, if there are concerns, that we're also looking at our practice… Again, it's just part of the expectation that we have is that we need to understand what's happening for the student, and then respond to that behavior. We do behaviors communication. So if you don't understand the why and what's happening, then you can't respond.”* [SpEd teacher]	Converged
Existing Supports	1.7 (1.1)	Low	*“I think a couple of things. Hugely important is time. We need planning time. We need so much time to really be able to sit again with curriculum, with goals, with expectations, with strategies, with tools, and figure out how it's all going to come together.”* [GenEd teacher]	Converged
EBP Integration	1.9 (1.0)	Low	*“It's something that we do naturally in our school. Many of our teachers are using it. It's something that we kind of really push of modeling in our schools, and so it's something we use a lot. It's something we talk about a lot. We've taken a lot of trainings on modeling specifically, and different types of modeling, and how to use modeling.”* [SpEd teacher]	Diverged
Recognition	2.0 (1.0)	Moderate	*“I would just like to hear more positive things in general, it would be nice if they recognized that those things were happening and that they were benefiting her. I think that it's often taken for granted. And so it doesn't get recognized as like, "Wow, you really are putting these systems into place and keeping them going. And that's why things are smooth."*[SpEd teacher]	Converged
Educational Supports	2.0 (1.7)	Moderate	*“Would love a budget to pay for all the things. It costs money for reinforcement. Would you like to see my Amazon receipts? Of course I would love it if I had some sort of budget to do this, but I'm also the type of educator that I'm just going to do it anyways because it's the right thing. And it works for some kids, maybe not others, but it definitely does. And so I just do it because I know it's the right thing. But it would be great if I had a reinforcement budget.”* [SpEd teacher]	Converged
Focus on EBP	2.4 (0.9)	Moderate	*“I think a lot of General Ed teachers don't have a clue what we're doing in this class, because it's not a collaborative school, it's not an inclusive school. And so I think the times are changing where they'll know more of what we're doing, because it's going to be an expectation that they do and it will be more team teaching. But as of now, I don't think they really have a clue, all the work that we do.”* [SpEd teacher]	Diverged
**Total SICS**	1.8 (0.8)			

Overall, quantitative results indicated that implementation climate constructs were rated low to moderate by educators. These findings often converged with qualitative data which illustrated a lack of several important implementation climate factors within general education settings in schools. Rewards for EBP use were rated lowest by participating educators. This was consistent with qualitative findings, as educators primarily expressed a lack of interest in receiving tangible rewards for using EBPs or did not discuss rewards at all. Qualitative findings converged with quantitative data and provided greater insight into why both existing and educational support for EBP use may have been rated low quantitatively. Educators reported that existing preparation time was not intended to be used to discuss or prepare for EBP implementation. Although integration of EBPs was rated low quantitatively, qualitative data highlighted that three of the four EBPs were relatively well integrated into their schools, illustrating a divergence in quantitative and qualitative data. This was particularly true for EBPs that fit more seamlessly into existing systems (e.g. reinforcement and positive behavior support systems), compared to EBPs that would require educators to learn a new intervention and perhaps require more use of data and tracking student progress (i.e. ABI). Indeed, use of data to support EBP implementation also was rated low quantitatively and rarely discussed in interviews, suggesting a convergence of these two data strands. Finally, focus on EBP was rated highest by educators based on quantitative data. These findings are consistent with previous research, which found that this construct was rated highest across all climate subscales in schools [20]. However, qualitative data diverged from these findings as educators revealed a lack of focus on EBPs in their classrooms and schools. Similar to other climate subscales, educators often referred to the lack of time and training dedicated to improving EBP implementation, particularly for general educator teachers.

## Discussion

Findings from the current study provide further evidence regarding the theory that implementation climate is a malleable factor that should be addressed to improve implementation efforts [23]. Moreover, these findings highlight the potential importance of tailoring implementation strategies to improve implementation climate within general education settings. By leveraging mixed-methods analyses, our findings provide a nuanced understanding of what the implementation climate in schools looks like in relation to implementing EBPs for autistic youth. Previous studies have begun to explore the implementation climate within educational settings. These studies have consistently indicated that positive implementation climate is associated with educator job satisfaction, positive student outcomes, and improved fidelity to EBPs in school settings [19, 37, 38]. Additionally, recent work suggests that implementation climate may be particularly important for educators and providers who have less experience working with autistic populations [22]. Research also has illustrated the positive relationship between implementation leadership and implementation climate [19] and outlined several strategies that school leaders may use in order to foster school climates that are supportive of implementation efforts (e.g. supporting teachers in obtaining professional development opportunities, allocating resources to EBP implementation, providing public recognition for educators’ EBP knowledge and implementation efforts) [26].

Findings from the current study also described educator perspectives regarding constructs related to implementation climate within their schools and revealed several areas for improvement. These findings align with previous work suggesting that using implementation strategies that are selected and tailored to address specific barriers to implementation may be particularly effective for improving implementation efforts, though further research evaluating the effectiveness of tailored strategies is needed [39]. Based on these findings, we outline several recommendations to tailor implementation strategies to improve implementation climate in general educational settings.

### Recommendations for tailoring implementation strategies

Mixed-methods analysis highlighted findings that are consistent with previous research evaluating implementation climate in schools. For example, rewards were not used frequently and suggest a need for reevaluating the kinds of rewards used within educational settings specifically [20]. While Lyon et al. [20] found that recognition for EBP use was rated in the good range, our study found that recognition was rated low by educators. Qualitative findings suggested that educators are primarily receiving informal verbal recognition. Overall, these findings suggest that it may be beneficial to consider the use of rewards and recognition that are more tailored for school settings specifically, and that will be perceived as positive or meaningful by educators given that some educators reported feeling uncomfortable about receiving recognition. Participant responses indicate that providing educators with additional preparation time and providing more formal opportunities for educators to receive recognition from leadership and their colleagues may be particularly helpful in this context.

Findings also suggest that repurposing and tailoring existing preparation and meeting time to specifically increase communication and collaboration across educators around EBP use may be beneficial. Communication strategies may include outlining lesson plans and intervention strategies for the week, collaborative group meetings or online asynchronous support for educators, using implementation diaries, and opportunities to communicate and share implementation successes. In terms of educational support, educators revealed a gap in training for general education teachers specifically. While it is imperative not to burden educators with excess work and mandatory training, it may be important to offer professional development or training opportunities related to supporting autistic students in general education settings. General education teachers play a key role on the education teams for autistic students in inclusive settings, but findings from this study suggest that they are not offered the same professional development opportunities needed to best support their autistic students. Future implementation efforts should ensure that these opportunities are afforded to them.

Lastly, findings related to the integration and focus on EBPs within general education settings, as well as use of data to support EBP use highlighted a number of areas for improvement. Firstly, findings suggest that interventions involving the use of data may require more time and training in order to be integrated more effectively in school settings and better tailored for educators. Given the increased push for inclusion of autistic students in their least restrictive environment, it is imperative that general education teachers are offered the opportunity to obtain additional training related to EBPs and how to tailor them for their autistic students.

This study provides further evidence that implementation climate is an important construct to support in schools. Overall, quantitative and qualitative data reveal that the implementation climate in general education settings may be suboptimal for implementing EBPs for autistic students and findings consistently revealed the need for additional training and for improved tailoring of implementation efforts. Future research must consider that an implementation climate most conducive to autism EBP implementation may look different as compared to previous studies examining implementation climate in schools.

### Limitations and future directions

Several limitations should be considered when interpreting the findings in this study. First, data collection occurred during the height of the COVID-19 pandemic. During this time, schools faced unprecedented challenges which may have impacted educators’ perception of their implementation climate not reflective of typical inclusive settings. The rapid shift to virtual learning environments [40] and reallocation of resources [41] in response to the COVID-19 pandemic presented challenges to general education inclusion, EBP use, and EBP adaptations. However, teachers continued to use EBPs through parent education and dropping materials off at students’ homes. Despite this creative problem-solving, the challenges educators faced may have affected their responses regarding educational support, recognition and rewards for EBP use, and existing support to deliver EBPs for autistic students.

Second, there are several outer context variables that were not evaluated in this study. In addition to the long-term impact of the COVID-19 pandemic, outer contextual factors such as funding for educational programs, changing educational policies in the United States, and students’ home environments likely all have a significant impact on both the implementation climate within schools as well as student-level outcomes. In order to truly understand factors influencing implementation climate and strategies tailored to improve implementation climate, future research should explore the impact of outer contextual factors on these domains.

Third, although our participant sample reflected the demographics within the US State in which the data were collected, it primarily consisted of educators who identified as white, limiting the generalizability of our findings to schools with increased racial and ethnic diversity. General education settings with more diverse staff compositions may reflect implementation climate dynamics not captured in our study due to cultural and contextual factors. To better understand how implementation climate varies across different school settings, future work should include more diverse educator samples.

Fourth, there is variation in the number of autistic students supported by participating educators. Some educators worked with one autistic student, while others supported multiple students. This variation may impact how educators perceive and report their experiences. Educators working with more than one student may provide observations that reflect general patterns across their caseload, while those working with one student may provide observations specific to that individual student Consequently, differences in the number of students educators work with may have influenced their experiences, which limits generalizability. Future work would benefit from stratifying samples based on experience or the number of students educators support to account for these factors.

Lastly, our study did not directly measure child-level outcomes in relation to implementation climate, limiting our understanding of the impact variation in implementation climate may have on student success. Future research should incorporate direct measures of child-level outcomes for autistic children to identify how implementation climate significantly impacts educational and behavioral outcomes of autistic students. Results from these studies also could inform which constructs of implementation climate to prioritize when optimizing resource allocation in publicly funded school systems. Additionally, we did not examine child-level outcomes in relation to the home environment, restricting our ability to evaluate how factors outside of the school setting may impact autistic student success as this study solely focuses on the school context. Previous work has highlighted the utility of an implementation strategy designed to support both caregivers and teachers of autistic students, that may be relevant when considering implementation climate in schools [42]. Subsequent studies should continue to evaluate how the home environment contributes to educational and behavioral outcomes for students as well. A more comprehensive view across settings may provide a deeper insight into the external factors and supports that most promote autistic students’ success.

## Conclusion

Previous research has sought to improve implementation climate by first addressing implementation leadership [43] with the ultimate goal of increasing provider fidelity. Additional studies have further evaluated the impact of both implementation leadership and climate on fidelity in elementary schools. Findings from these studies indicate that positive organizational culture and climate are associated with higher fidelity. Findings also support the notion that stronger implementation leadership predicts higher implementation climate, highlighting the malleability of this key factor in implementation success [43, 44]. However, the current study illustrated several areas for improving implementation climate school settings, and highlighted the importance of tailoring implementation strategies so that they are best suited for the educational context. There are implementation strategies that strategically focus on developing and supporting implementation climate that ought to be tested in special education contexts [45, 46]. Future research must consider how to best tailor EBP training to focus on supporting autistic students and address gaps in knowledge for general education teachers. Implementation efforts that focus on implementation climate dimensions may be particularly critical for supporting EBP use and sustainment in schools. It is critical that schools foster a conducive implementation climate to support EBP installation and sustained implementation to ensure autistic students are supported and included to the maximal extent possible in their least restrictive environment.

## Supplementary Information

Supplementary Material 1.

Supplementary Material 2.

## Data Availability

The data described in this manuscript is freely available. Please contact the lead author for more information.

## Abbreviations

CFIR: Consolidated Framework for Implementation Research
EBPs: Evidence-based practices
EPIS: Exploration, Preparation, Implementation, Sustainment
IDEA: Individuals with Disabilities Education Act
LRE: Least restrictive environment
Quan: Quantitative data
QUAL: Qualitative data
SICS: School Implementation Climate Scale
