# Supplementary material for: Unpacking the implementation climate in general education settings in public schools: a sequential-explanatory mixed-methods study
Source: Implement Sci Commun. 2025 Nov 28;6:132. doi: 10.1186/s43058-025-00810-0 (PMC12661666; doi:10.1186/s43058-025-00810-0)
Supplement: Supplementary file 1 — Supplementary Material 1. [file 43058_2025_810_MOESM1_ESM.docx]

**Supplemental Material**

*CORE-Q*

| Domain 1: Research Team and Reflexivity | Guide questions/description | Present |
| --- | --- | --- |
| Personal Characteristics |  |  |
| 1. Interviewer/Facilitator | Which author/s conducted the interview or focus group? | **Y** |
| 1. Credentials | What were the researchers’ credentials? E.g., PhD, MD | **N** |
| 1. Occupation | What was their occupation at the time of the study? | **Y** |
| 1. Gender | Was the researcher male or female? | **N** |
| 1. Experience and training | What experience or training did the researcher have? | **Y** |
| Relationship with Participants |  |  |
| 1. Relationship established | Was a relationship established prior to study commencement? | **N** |
| 1. Participant knowledge of the interviewer | What did the participants know about the researcher? E.g., personal goals, reasons for doing the research | **N** |
| 1. Interviewer characteristics | What characteristics were reported about the interviewer/facilitator? E.g., Bias, assumptions, reasons and interests in the research topic | **N** |
| Domain 2: Study Design |  |  |
| 1. Methodological orientation and Theory | What methodological orientation was stated to underpin the study? E.g., grounded theory, discourse analysis, ethnography, phenomenology, content analysis | **Y** |
| 1. Sampling | How were participants selected? E.g., purposive, convenience, consecutive, snowball | **Y** |
| 1. Method of approach | How were participants approached? E.g., face-to-face, telephone, mail, email | **Y** |
| 1. Sample size | How many participants were in the study? | **Y** |
| 1. Non-participation setting | How many people refused to participate or dropped out? Reasons? |  |
| 1. Setting of data collection | Where was the data collected? E.g., home, clinic, workplace | **Y** |
| 1. Presence of non-participants | Was anyone else present besides the participants and researchers? | **N** |
| 1. Description of sample | What are the important characteristics of the sample? E.g., demographic data, date | **Y** |
| Data collection |  |  |
| 1. Interview guide | Were questions, prompts, guides provided by the authors? Was it pilot tested? | **N** |
| 1. Repeat interviews | Were repeat interviews carried out? If yes, how many? | **N** |
| 1. Audio/visual recording | Did the research use audio or visual recording to collect the data? | **Y** |
| 1. Field notes | Were field notes made during and/or after the interview or focus group? | **N** |
| 1. Duration | What was the duration of the interviews or focus groups? | **Y** |
| 1. Data saturation | Was data saturation discussed? | **Y** |
| 1. Transcripts returned | Were transcripts returned to participants for comment and/or correction? | **N** |
| Domain 3: Analysis sand findings |  |  |
| 1. Number of coders | How many data coders coded the data? | **Y** |
| 1. Description of the coding tree | Did authors provide a description of the coding tree? | **Y** |
| 1. Derivation of themes | Were themes identified in advance or derived from the data? | **Y** |
| 1. Software | What software, if applicable, was used to manage the data? | **Y** |
| 1. Participant checking | Did participants provide feedback on the findings? | **N** |
| Reporting |  |  |
| 1. Quotations presented | Were participant quotations presented to illustrate the themes/findings? Was each quotation identified? E.g., participant number | **Y** |
| 1. Data and findings consistent | Was there consistency between the data presented and the findings? | **Y** |
| 1. Clarity of major themes | Were major themes clearly presented in the findings? | **Y** |
| 1. Clarity of minor themes | Is there a description of diverse cases of discussion of minor themes? | **Y** |
