# Supplementary material for: Unpacking the implementation climate in general education settings in public schools: a sequential-explanatory mixed-methods study
Source: Implement Sci Commun. 2025 Nov 28;6:132. doi: 10.1186/s43058-025-00810-0 (PMC12661666; doi:10.1186/s43058-025-00810-0)
Supplement: Supplementary file 2 — Supplementary Material 2. [file 43058_2025_810_MOESM2_ESM.docx]

Supplemental Material.. Qualitative Codebook

| **Category/Code** | **Definition** |
| --- | --- |
| **Outer Setting** | Any mention factors related to the broader context in which the school exists (e.g. district, state), including: the IDEA policy driving LRE and in the inclusion policy. |
| **Inner Setting** |  |
| School | Any mention of the factors associated with the school setting in which the innovation is being implemented, including: IEP, UDL, school culture around inclusion, school culture around neurodiversity, admin trust, support/appreciation, and relationships (or lack thereof), PBIS/reinforcement systems, implementation climate and implementation leadership, turnover, top-down approaches to programming, shifts in the school culture, ICS-EBP use, SpEd programming, and the MTSS framework. Discussion related to training and support for training should fall under school. EBP is being integrated more at the school level (e.g. into curricula, by leadership, by multiple educators and in several contexts or settings)/is becoming a school-wide practice. Discussions of educators supporting/helping each other’s students would be coded under communication & collaboration. Discussion of communication with/from leadership would be coded under communication and collaboration. |
| Classroom Placement | This code refers to factors related to classroom placements within the school, including general education inclusion and decisions around inclusion, adjusting the EBP to the classroom environment, challenges (e.g. teasing/bullying) and/or support in GenEd classrooms, classroom supports (e.g. structure, timing, environmental supports), tailoring the classroom to students, composition of the classroom, reactive/proactive putting out fires in classroom, and the reverse inclusion model. |
| Resources | This code refers to resources within the inner setting that may impact implementation, including: funding, procurement of resources, lack of resources/resource availability for high-need students, teaching resources (e.g. curricula resources), time (e.g. to communication, use the EBP, co-teaching model), access to specialists (e.g. speech/OT), paras performing duties outside of job responsibilities, lower caseload, and making supplies (e.g. visuals), paraeducator challenges (e.g. high caseloads, low morale). |
| **Individuals** |  |
| Child Characteristics for Inclusion/Appropriateness and Fit for Student Inclusion | Any mention of individual (student) characteristics impacting students’ fit/appropriateness for inclusion including peers, placement, inclusion factors (e.g. decision-making, encouragement, lack of breaks, level of support needs, level of independence, “mean” kids in the inclusion setting, push in/pull out fit), and characteristics including: sociable/friendly, distracted, academically on tasks, completing work, communication needs, collaboration, motivation, personality, and preference for reverse inclusion. |
| **Implementation Process** |  |
| Educator Characteristics and Considerations for EBP Use | Characteristics of the individuals involved in the implementation process including their capability (e.g. knowledge and skills related to the EBP/population), needs (e.g. additional training/knowledge), biases towards students, motivation around implementation, opportunity to engage in implementation, knowledge and excitement for creating resources, relationship with students that helps with using the EBP (e.g. offering choices, familiarity with the students’ needs, knowledge of managing their behaviors), tailoring/adapting the EBP, tailoring teaching styles, evaluating the impact of the EBP on student progress and compliance, flexibility, attitudes about the EBP, intentionality and sense of responsibility about EBP use, following EBP steps, and advocacy for using the EBP. All characteristics should be in relation to receipt/use of the EBP. |
| Educator Characteristics and Considerations for EBP Use: Differences and Similarities between and within GenEd and SpEd Educators (including Paras) | This code broadly covers differences and similarities between educators, including differences or similarities in knowledge (e.g. GenEd teachers don’t know what paras/SpEd teachers know). This may include different philosophies or attitudes related to interventions/strategies. |
| Family Characteristics | This code includes characteristics of the innovation recipients (families) including schools needing to have more support available for families, student-family dynamics, and family resources/support. Factors related to the activities/strategies used to implement the innovation, including assessing needs, planning, engaging, reflecting/evaluating, and adapting. This code focuses on the relationship between families and schools, how EBP use is impacted by parental input/involvement, lack of transparency between the school and parents, and parent advocacy in relation to receipt/use of the EBP. This code will refer to ANY families, not just the family of the included student with autism. |
| Child Characteristics and Response to EBP use | Factors related to the activities/strategies used to implement the innovation, including assessing needs, planning, engaging, reflecting/evaluating, and adapting. This code broadly covers characteristics of any students including challenging behaviors, level of support and attention needed, sensory stimulation, independence/autonomy/self-advocacy, communication needs/skills, doing well in GenEd, integration with non-autistic peers/sense of belonging, and use of tools (eg visuals, checklists), in relation to receipt or use of the EBP. This code also covers fit with the EBP (e.g. matches level of support needed, fit of intervention for educational needs, fit of resources, time of day that support is needed), student response to the EBP, information from IEP informing EBP use, preference for the classroom is respected, and specific instruction in relation to receipt/use of the EBP. |
| Communication and Collaboration across and within teams | This code broadly covers communication and collaboration throughout the implementation process, including: team communication across levels, having dedicated time to communicate, planning/oversight/troubleshooting, leadership communication and receptivity (or lack), liaison between GenEd/SpEd, ability to talk to “main person” throughout the day, staff on the same page, strong communication among colleagues, frequent/daily communication, formal/informal communication, monitoring student behavior/evaluating data or feedback, reflection, increased discussion around EBP use and discussing EBP at staff meetings, frequent and proactive communication, MTSS team to discuss specific needs. Collaboration across educators and teams to as part of the implementation processing, including: co-teaching/push-in time, positive attitudes towards the team, similar training across team, disconnect across teams, collaborating across grades and opportunities to collaborate throughout the day, inconsistent OR consistent responses/delivery of EBP across teams for the same students, and empathy among the teams. This includes examples of educators providing support to other educators’ students or using the EBP for other students (with or without being asked). This also includes any discussion of LACK of communication and/or collaboration. |
| **The WHAT Innovation Characteristics** | Any mention of characteristics related to the EBP/Intervention including helping students identify procedures/guidelines, supporting students (e.g. socially, academically, increasing autonomy), being slow to use the EBP, the adaptability of the EBP, can be integrated or used with other practices/strategies, visual schedules, alignment with grade level expectations, EBP being effective, EBP is natural to use/straightforward, modeling of the EBP, and being able to multi-task. |
